# Different modulation of *Panax notoginseng* on the absorption profiling of triptolide and tripterine from *Tripterygium wilfordii* in rat intestine

**DOI:** 10.1186/s13020-017-0157-6

**Published:** 2018-01-08

**Authors:** Yiqun Li, Huiting Cao, Mengzhu Liu, Benyong Zhang, Xinlong Zhang, Donglei Shi, Liwei Guo, Jinao Duan, Xueping Zhou, Huaxu Zhu, Qichun Zhang

**Affiliations:** 1 Jiangsu Collaborative Innovation Center of Chinese Medicinal Resources Industrialization, Nanjing University of Chinese Medicine, Nanjing, 210023 China; 2 Jiangsu Key Laboratory for High Technology Research of TCM Formulae, Nanjing University of Chinese Medicine, Nanjing, 210023 China; 3 Department of Pharmacology, School of Pharmacy, Nanjing University of Chinese Medicine, Nanjing, 210023 China

**Keywords:** Single-pass intestinal perfusion, Qing-Luo Tong-Bi, Effective permeability

## Abstract

**Background:**

Compatibility with *Panax notoginseng* (PN) reduced the plasma concentration of triptolide and delayed the T_max_ of *Tripterygium wilfordii* (TW), the sovereign medicine of Qing-Luo Tong-Bi decoction, which hinted the absorption process of triptolide might be involved in decreasing the toxicity in liver and kidney.

**Methods:**

The absorption of triptolide, triptonide, wilforlide and tripterine from monomer, TW, TW-PN, TW-*Caulis Sinomenii* (TW-CS) and Qing-Luo Tong-Bi were analyzed in duodenum, jejunum, ileum and colon of rat via single-pass intestinal perfusion model. An UPLC-MS/MS analysis method was developed to determine the concentration of triptolide, triptonide, wilforlide and tripterine in the inlet and outlet. Then P_eff_, 10 cm%ABS and K_a_ were calculated based on the perfusate flux, perfusate volume and candidate chemicals concentration.

**Results:**

The absorption of triptolide, triptonide, wilforlide and tripterine in duodenum, jejunum, ileum and colon was independent of concentration within range of 3–9 μg/mL. The target compounds, triptolide, triptonide, wilforlide and tripterine from the TW extract, showed higher absorption extent and rate than those administrated alone, and compared with the absorption situation of the chemicals of TW extract, the absorption of triptolide, triptonide and wilforlide of the extract of TW-PN, TW-CS and Qing-Luo Tong-Bi were decreased in these intestinal segments. However, PN-promoted tripterine absorption was observed in the intestine.

**Conclusions:**

Modulation of absorption of chemicals in TW by subsidiary herbs may be responsible for reinforcing the actions and neutralizing the adverse effects through compatibility in the formula of Qing-Luo Tong-Bi. PN inhibits the absorption of triptolide of TW and promote the absorption of tripterine.

**Electronic supplementary material:**

The online version of this article (10.1186/s13020-017-0157-6) contains supplementary material, which is available to authorized users.

## Background

*Tripterygium wilfordii* (TW), the root of the herb *Tripterygium wilfordii* Hook. F., is a routine and important traditional Chinese herbal medicine, the properties and actions of which are described as dispelling pathogenic wind and removing dampness, promoting blood circulation and freeing meridians, detumescence for suppressing pains, destroying parasites and detoxifying [1, 2]. Clinically, TW is employed primarily to treat disorders associated with autoimmunity and inflammation such as rheumatoid arthritis, systemic lupus erythematosus, nephritis, encephalomyelitis and psoriasis [1, 3, 4]. Due to the narrow therapeutic window and adverse effects such as hepatotoxicity and nephrotoxicity emerge usually during the therapeutic process [5]. Compatibility is the principal protocol of medicines application in traditional Chinese medicine, the aim of which is to reinforce the expected curative effect and neutralize the toxicity of sovereign medicine in a prescription [6].

Qing-Luo Tong-Bi (QLTB) decoction is a prevalent formula including TW, *Panax notoginseng* (PN), *Caulis Sinomenii* (CS), *Rehmannia glutinosa* (RG) and *Bombyx batryticatus* (BB) in a proportion of 15:3:15:15:10 from the Rheumatism Department of Jiangsu Province Hospital of Traditional Chinese Medicine (Nanjing, PRC). In the previous investigation [6], PN was shown to significantly block the TW-induced elevation of alanine aminotransferase (ALT), aspartate aminotransferase (AST) and lactate dehydrogenase (LDH) in the rat plasma and ameliorate the histopathological damage of liver. Further pharmacokinetics analysis of triptolide, one of key active components of TW, demonstrated that PN changed the pharmacokinetic process of triptolide. With bare change of the dose in the body, extended T_max_ and decreased C_max_, one of the possible mechanisms for PN to interfere the pharmacokinetic process of triptolide is proposed to modulate the absorption in intestinal tract. Meanwhile, the absorption of other chemicals from TW is still vague with or without PN.

Absorption is a complex kinetic process described via several in vitro or in vivo models. Single-pass intestinal perfusion (SPIP) technique is a powerful predictive absorption model developed to provide mathematical descriptions of rate and extent of drug absorption in vivo [7]. Unlike the cell model as Caco-2, SPIP has advantage properties of having intact blood supply and the ability to perform a mechanism evaluation of absorption process under controlled and semi conscience conditions. The effective permeability coefficient (P_eff_) is directly related to the first-order absorption rate process in this model, which is used to estimate the extent of absorption [8]. Moreover, SPIP has been demonstrated to closely correlate to in vivo human data and a useful tool to predict absorption for both passive and carrier-mediated transport [9, 10].

To achieve more comprehensive understanding the absorption properties of TW under combination of the subsidiary herbal medicines, four representative components including triptolide, triptonide, wilforlide and tripterine are adopted to investigate the absorption parameters via SPIP model in rat. UPLC-MS/MS method was also developed to analyse the concentration change of drugs in the intestinal perfusate solution inlet and outlet. The results demonstrate that the absorption of triptolide, triptonide and wilforlide is inhibited in the formulae of TW-PN, TW-SC and QLTB. The influx of tripterine in the intestine is elevated in TW-PN but still decreased in TW-SC. With synergic influence of PN and SC, the absorption of tripterine in QLTB is increased in duodenum and jejunum, and reduced in ileum and colon.

## Methods

The Minimum Standards of Reporting Checklist (Additional file 1) contains details of the experimental design, and statistics, and resources used in this study.

### Chemicals and reagents

**Fig. 1:**
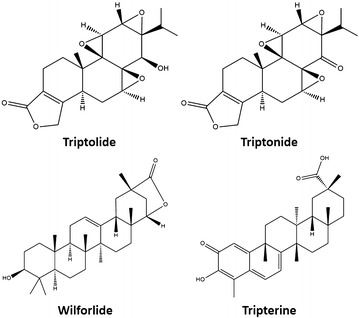
Chemical structure of triptolide, triptonide, wilforlide and tripterine

Triptolide (purity: 98%, Batch Number: 111567–200603) and prednisolone (purity: 99.4%, Batch Number: 100153–201004) were purchased from the National Institute for the Control of Pharmaceutical and Biological products (Beijing, China). Triptonide (purity: 98%, Batch Number: 20120720), wilforlide (purity: 98%, Batch Number: 20120720), tripterine (purity: 98%, Batch Number: 20120720) were purchased from Nanjing Zelang Medical Technology Co., Ltd. (Nanjing, China). The chemical structures of triptolide, triptonide, wilforlide and tripterine were shown in Fig. 1. TW was purchased from Xichang Materials Company of Sichuan (Batch Number: 120620), while PN was collected at Bozhou Medicine Company of Anhui (Batch Number: 111208), and all herbal medicine were identified by Dr. Qinan Wu (Department of Pharmacognosy, Nanjing University of Chinese Medicine, Nanjing, China) (College of Pharmacy, Nanjing University of Chinese Medicine, Nanjing, China). Normal saline was purchased from Nanjing Bianzheng Medical Technology Co., Ltd. (Nanjing, China). Acetonitrile and methanol used for UPLC were chromatographic grade (Merck, Darmstadt, Germany). All of the other reagents were analytical grade (Sino Pharm Chemical Reagent Co., Ltd., Shanghai, China). Milli-Q water (Millipore, Bedford, MA, USA) was used throughout the study.

### Apparatus

These primary apparatus utilized in present research include BT100-1L constant flow peristalsis pump (Longer Pump Co., Ltd.), UPLC AcquityTM system (Waters, USA), Xevo triple quadrupole mass spectrometer (Waters, USA), Electrospray ion source (ESI), Masslynx 4.1 (Waters, USA), Votex Genius 3 vortex mixing equipment (Germany IKA company), Allegra 64 r Centrifuge including a high-speed refrigerated centrifuge (Beckman Coulter, USA), Ultra-pure water system (Millipore, USA), Multifuge X1R refrigerated centrifuge (Thermo, USA), Votex Genius 3 vortex mixing equipment (Germany IKA company), FE20 pH meter (Mettler-Toledo) and CentriVap^®^ centrifugal concentrator (Labconco, USA).

### Preparation of the extracts of TW, TW-PN, TW-SC and QLTB

Herb material of TW (3000 g) were crushed to pieces and were extracted with boiling water (1:11, w/v, and then 1:7, w/v) for 1.5 h each time. The filtrates filtered through gauze were merged and evaporated to approximately 150 mL under a vacuum at 65 °C by rotary evaporation. Then, the concentrate of TW was obtained and stored at 4 °C until use. A total of 3600 g of TW-PN with pieces of TW (3000 g) and PN (600 g), TW-SC of 6000 g with pieces of TW (3000 g) and CS (3000 g) and QLTB of 11600 g with pieces of TW (3000 g), PN (600 g), CS (3000 g), RG (3000 g), BB (2000 g) were extracted through the same process of TW product.

### Preparation of perfusate

The stock solution of triptolide (100 μg/mL), triptonide (100 μg/mL), wilforlide (500 μg/mL), tripterine (500 μg/mL) and internal standard prednisolone (300 μg/mL) were prepared by dissolving accurately weighed chemical into methanol respectively. The perfusate solutions of these chemicals were prepared by diluting the corresponding stock solutions with HBSS solution (CaCl_2_, 0.14 g; d-glucose, 4.5 g; NaCl, 9.164 g; HEPES, 5.96 g; KCl, 0.4 g; NaHCO_3_, 0.37 g; MgSO_4_·7H_2_O, 0.245 g; Na_2_HPO_4_·12H_2_O, 0.126 g; KH_2_PO_4_, 0.06 g; dissolved in water to 1 L, pH 7.4) to concentrations of 3, 6, 9 μg/mL.

The four extracts of TW, TW-PN, TW-CS and QLTB were freeze-dried into powder and redissolved in HBSS solution to obtain TW, TW-PN, TW-CS and QLTB perfusate solutions and stored at 4 °C until use. The concentrations of triptolide in TW, TW-PN, TW-CS and QLTB working solutions were regulated to 3 μg/mL. All the intestinal perfusate solutions were stored at 4 °C until use.

### Animal experiment and samples collection

Male Sprague–Dawley rats (250 ± 15 g) were supplied by Nanjing University of Chinese Medicine Animal Center and kept in a breeding room with temperature of 24 ± 2 °C, humidity of 55 ± 5%, and 12 h light/dark cycle for 7 days before the experiment. Animal welfare and experimental procedures were strictly in accordance with the Guide for the Care and Use of Laboratory Animals (US National Research Council, 1996). The study protocol and the total number of rat were approved by the Animal Care and Use Committee of Nanjing University of Chinese Medicine. The investigation conformed to the Guide for the Care and Use of Laboratory Animals published by the US National Institutes of Health (NIH Publication No. 85-23, revised 1996). Forty-eight rats were randomized into eight groups (Triptolide, Triptonide, Wilforlide, Tripterine, TW, TW-PN, TW-CS and QLTB, n = 6 for each group).

Rats were fasted for 16 h with free access to water prior to the perfusion study and anesthetized with 10% chloral hydrate solution (3.4 mL/kg, i.p.). A laparotomy was made through a midline incision of about 4 cm to separate the duodenum, jejunum, ileum and colon from the abdominal cavity, and approximate 10 cm of the four intestinal segments were exposed. At both ends of each measured intestinal segments, silicone cannulas were inserted. First, the intestinal lumen was cleaned by normal saline (37 °C) perfusion via the inlet until the effluent from the outlet was judged to be free of feces and clear. The perfusion solution was pumped by peristaltic pump through the intestine at a flow rate of 1.0 mL/min for 10 min. Following that, the perfusion solution through the intestinal lumen was changed to a constant flow rate of 0.2 mL/min for 20 min. After being stable for 30 min, the effluent solution were collected from the outlets of the four intestinal segments during each 10-min period (30–40, 40–50, 50–60, 60–70, 70–80 min) into pre-weighted vials. Then the weight of each perfusion solution was analyzed. During the perfusion operation, these exposed intestines were covered with gauze that had been moistened by frequent applications of warm (37 °C) normal saline, and kept warm by a small lamp placed over the area. At the end of the sampling, animals were euthanized with saturated potassium chloride solution by intracardiac injection, according to protocols of euthanasia in experimental animals. After death, the four segments of intestine were removed for measurements of length and radius (l and r, respectively). Duodenum segment was measured from 1 cm blow the pylorus, jejunum segment was measured from 15 cm blow the pylorus, ileum segment was measured from 20 cm above the cecum and colon segment was measured from 1 cm blow the cecum. The constant length of segment is 10 cm.

### Sample preparation

The intestinal perfusion solution samples for UPLC-MS/MS analysis were prepared as follows. The internal standard prednisolone (100 μL, 30 μg/mL) was added to intestinal perfusion solution samples (1.0 mL) in a 10.0 mL centrifuge tube. After vortex with 4 mL ethyl acetate for 3 min, the intestinal perfusion solution samples were centrifuged at 3000 rpm for 10 min and the supernatants was transferred into a new 10.0 mL tube. Then another 4 mL ethyl acetate was added to the residue followed by vortex for 3 min and centrifuged at 3000 rpm for 10 min. The two supernatants were merged and evaporated to dryness by the centrifugal concentrator at 40 °C. The residue was dissolved by 1.0 mL acetonitrile followed by vortex for 3 min and centrifuging at 12,000 rpm for 10 min. At last, 20 μL of the supernatant was injected into the UPLC-MS/MS system for analysis.

### UPLC conditions and UPLC-MS/MS analysis

An Agilent Zorbax Eclipse Plus C18 column (2.1 mm × 100 mm, 1.7 μm, Agilent, USA) was employed and the column temperature was kept at 35 °C. Mobile phase A consisted of acetonitrile and mobile phase B was 0.1% formic acid (v/v) in water. The gradient conditions were as follows: 0–1 min, 0–35% A; 1–2 min, 35–85% A; 2–4 min, 85% A; 4–4.8 min, 85–98% A; 4.8–5 min, 98–35% A. The injection volume was 2 μL and the flow rate of 0.4 mL/min.

For MS detection, we used an electrospray ionization source operating in the positive ion mode. The scanning mode we used was multiple reaction monitoring (MRM). The ion source temperature was set at 110 °C. A desolvation gas temperature of 350 °C, a cone gas rate of 50 L/h and a desolvation gas flow of 1000 L/h were used. The capillary voltage and cone voltage were set at 3000 and 40 V, respectively. The collision energy of prednisolone, triptolide, triptonide, wilforlide and tripterine are 40, 40, 40, 38 and 38 eV. Leucine-enkephalin was used as the lockmass in all analyses (m/z 556.2771 for positive ion mode and m/z 554.2615 for negative ion mode) at a concentration of 0.5 μg/mL with a flow rate of 5 μL/min. Data were collected in the centroid mode from m/z 100 to m/z 1000. The m/z of prednisolone, triptolide, triptonide, wilforlide and tripterine are as follows: 358.9/91.06, 360.89/91.07, 360.90/147.10, 451.03/201.07, 455.10/119.14.

### Method validation

The methods for quantitative analysis of triptolide, triptonide, wilforlide and tripterine in perfusate samples were validated according to the requirement of biopharmaceutical analysis, which was examined for specificity, linearity, precision, extraction recovery and stability under the UPLC analytical conditions.

The specificity was evaluated by comparing blank perfusate, perfusate mixed by triptolide, triptonide, wilforlide and tripterine, TW perfusate, TW-PN perfusate, TW-CS perfusate, and QLTB perfusate.

The precision was determined from inter-day and intra-day using five sets of quality control (QC) samples and was expressed by the relative standard deviation (RSD %), which was estimated as follows: RSD (%) = (standard deviation (SD)/the observed concentrations of replicate analyses of QC samples (Cobs)) × 100. The QC samples of triptolide, triptonide, wilforlide and tripterine were diluted by those stock solutions in HBSS solution (pH 7.4) to produced three QC samples of each chemical as follows: triptolide (0.5, 1, 3 μg/mL), triptonide (0.5, 1, 2.5 μg/mL), wilforlide (0.2, 1, 2 μg/mL) and tripterine (0.2, 1, 2 μg/mL).

The extraction recoveries were determined by calculating the ratio of triptolide, triptonide, wilforlide and tripterine detected in QC samples against that initial content in HBSS solution.

The stability of the method was evaluated by analyzing QC samples mixed with triptolide, triptonide, wilforlide and tripterine with concentrations of 2.5, 5, 2.5, 5 μg/mL at 37 °C for 0 and 2 h.

### Calculation

The absorption parameters of triptolide, triptonide, wilforlide and tripterine were calculated according to the methods as previously described [11, 12].

The effective permeability, P_eff_, in the SPIP studies was calculated by gravimetric method and the volume of perfusate was corrected and calculated by the following equation (Eq. (1)):

1 $$ {\text{P}}_{\text{eff}} = \, - {\text{Qln}}\left( {{\text{C}}_{\text{out}} /{\text{C}}_{\text{in}} *{\text{Q}}_{\text{out}} /{\text{Q}}_{\text{in}} } \right)/ 2\uppi{\text{rl}} $$

where Q = constant perfusate flux of the peristaltic pump (0.2 mL/min), C_out_ = outlet drug concentration, C_in_ = inlet drug concentration, Q_out_ = outlet perfusate volume of each intestinal segment during the 10-min period, Q_in_ = inlet perfusate volume of each intestinal segment during the 10-min period, r = radius of every intestinal segment (duodenum, jejunum, ileum, colon), l = actual length of every intestinal segment (duodenum, jejunum, ileum, colon).

The K_a_ was calculated through the equation below (Eq. (2)):

2 $$ {\text{K}}_{\text{a}} = {\text{ Q}}\left( { 1- {\text{C}}_{\text{out}} /{\text{C}}_{\text{in}} } \right)/\uppi{\text{r}}^{ 2} {\text{l}} $$

where Q = constant perfusate flux of the peristaltic pump (0.2 mL/min), C_out_ = outlet drug concentration, C_in_ = inlet drug concentration, r = radius of every intestinal segment (duodenum, jejunum, ileum, colon), l = actual length of every intestinal segment (duodenum, jejunum, ileum, colon).

The percentage of 10 cm intestinal absorption (10 cm%ABS) was calculated through the equation below (Eq. (3)):

3 $$ 10\;{\text{cm}}\% {\text{ABS }} = \, \left( { 1- {\text{Q}}_{\text{out}} /{\text{Q}}_{\text{in}} *{\text{C}}_{\text{out}} /{\text{C}}_{\text{in}} } \right)* 100\% $$

where C_out_ = outlet drug concentration, C_in_ = inlet drug concentration, Q_out_ = outlet perfusate volume of each intestinal segment during the 10-min period, Q_in_ = inlet perfusate volume of each intestinal segment during the 10-min period.

### Statistical analysis

The statistical analyses were performed by SPSS 16.0 (SPSS Inc., Chicago, USA). Statistical comparison of 10 cm%ABS was performed using Tukey tests and one-way ANOVA. All values are expressed as mean ± standard deviation. Means were assumed to be statistically significant when p < 0.05.

## Results

Method validation for the HPLC assay of triptolide, triptonide, wilforlide and tripterine in rat plasma.

**Fig. 2:**
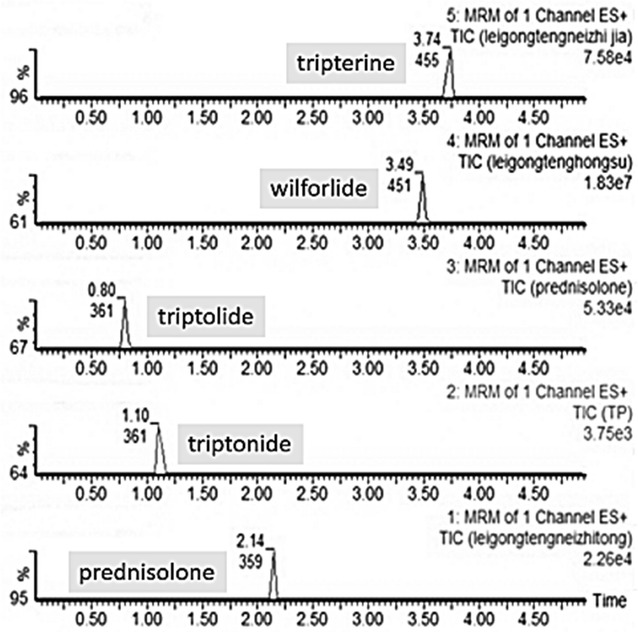
Representative chromatograms of triptolide, triptonide, wilforlide, tripterine and prednisolone

The retention times of triptolide, triptonide, wilforlide, tripterine and prednisolone were 1.59, 2.21, 3.63, 3.64 and 1.21 min, respectively. A significant endogenous peak could not be observed in this time-interval when these candidates were detected. Representative HPLC chromatograms are shown in Fig. 2.

The standard calibration curve of triptolide was linear over the range from 0.1 to 10.0 μg/mL with good linearity (R^2^ = 0.9990), and a typical equation for the calibration curve was Y = 0.0887X + 0.0026. The standard calibration curve of triptonide was linear over the range from 0.04 to 10.0 μg/mL with good linearity (R^2^ = 0.9976), and a typical equation for the calibration curve was Y = 0.1138X + 0.0108. The standard calibration curve of wilforlide was linear over the range from 0.02 to 20.0 μg/mL with good linearity (R^2^ = 0.9961), and a typical equation for the calibration curve was Y = 0.0232X + 0.0071. The standard calibration curve of tripterine was linear over the range from 0.02 to 5.0 μg/mL with good linearity (R^2^ = 0.9958), and a typical equation for the calibration curve was Y = 8.6242X + 2.9543.

The method showed good precision with intra-day and inter-day precision. As shown in Table 1 that the analytical precision of triptolide, triptonide, wilforlide and tripterine in intestinal perfusate solutions.

**Table 1:** Inter-day and intra-day precision of triptolide, triptonide, wilforlide and tripterine in intestinal perfusate solutions (inter-day n = 6; intra-day n = 6)

Con. (μg/mL)	Triptolide			Triptonide			Wilforlide			Tripterine		
	0.5	1.0	3.0	0.2	1.0	2.0	0.5	1.0	2.5	0.2	1.0	2.0
Inter-day (%)	1.11	0.52	1.62	1.63	0.40	1.27	1.48	0.95	1.11	1.53	0.33	1.14
Intra-day (%)	1.18	0.63	1.80	1.77	0.44	1.35	1.72	1.05	1.25	1.79	0.37	1.47

The extraction recoveries of triptolide were 82.75 ± 7.12, 85.36 ± 3.96 and 84.75 ± 4.11% at the concentrations of 0.5, 1.0 and 3.0 μg/mL. The extraction recoveries of triptonide were 78.60 ± 5.20, 74.80 ± 6.76 and 76.40 ± 5.78% at the concentrations of 0.5, 1.0 and 2.5 μg/mL. The extraction recoveries of wilforlide were 77.23 ± 7.04, 82.80 ± 5.86 and 82.68 ± 5.30% at the concentrations of 0.1, 1.0 and 2.0 μg/mL. The extraction recoveries of tripterine were 86.88 ± 7.67, 88.25 ± 4.62 and 84.69 ± 7.54% at the concentrations of 0.1, 1.0 and 2.0 μg/mL.

The stability analysis showed that the ratio of triptolide, triptonide, wilforlide and tripterine follow 2 h at 37 °C were 99.2, 96.86, 98.80 and 97.1% and showed no significant difference between the initial and tested concentrations.

The validated UPLC method was reproducible and reliable for determining triptolide, triptonide, wilforlide and tripterine in intestinal perfusate solutions.

### Absorption profiling of triptolide in intestinal tract

To analyze the basic absorption properties of triptolide in the small intestine and colon, the absorption parameters of P_eff_, 10 cm%ABS and K_a_ of triptolide at concentration of 3, 6 and 9 μg/mL were determined through SPIP model. As shown in Table 2, increasing the concentration of triptolide in the perfusate didn’t result in significant alteration of P_eff_, 10 cm%ABS and K_a_ (Table 2). Compared with pure triptolide, the P_eff_, 10 cm%ABS and K_a_ of triptolide in TW were enhanced in the whole intestine lumen. Meanwhile, pronounced increasing of P_eff_ and 10 cm%ABS were observed in the colon. Moreover, significantly elevated K_a_ of triptolide from TW was observed in jejunum and ileum. In TW-containing formulae, all the P_eff_, 10 cm%ABS and K_a_ of triptolide were decreased, and among which QLTB showed the maximum influence on the absorption of triptolide followed by TW-PN and TW-SC (Table 3).

**Table 2:** Absorption parameters of triptolide in perfusate solution within duodenum, jejunum, ileum and colon (${\bar{\text{x}}} \pm {\text{s}}$, n = 6)

Parameters	Con. (μg/mL)	Duodenum	Jejunum	Ileum	Colon
P_eff_ × 10^−3^	3	5.20 ± 1.72	4.98 ± 1.67	6.46 ± 2.0	6.35 ± 1.28
	6	5.05 ± 1.91	4.85 ± 1.88	6.36 ± 1.92	6.20 ± 1.45
	9	5.14 ± 0.56	4.77 ± 2.21	6.29 ± 2.31	6.36 ± 1.17
10 cm%ABS	3	42.7 ± 1.85	41.3 ± 2.4	47.8 ± 1.64	41.1 ± 3.33
	6	46.5 ± 3.52	41.6 ± 1.28	47.8 ± 1.34	41.8 ± 2.87
	9	45.8 ± 4.03	41.4 ± 1.67	48.4 ± 1.69	41.9 ± 2.88
K_a_ × 10^−2^	3	1.79 ± 0.33	1.64 ± 0.39	2.93 ± 0.29	1.81 ± 0.11
	6	1.75 ± 0.29	1.49 ± 0.79	2.86 ± 0.34	1.77 ± 0.23
	9	1.69 ± 0.31	1.53 ± 0.84	2.93 ± 0.24	1.69 ± 0.29

**Table 3:** Absorption parameters of triptolide of various formulae in perfusate solution within duodenum, jejunum, ileum and colon (${\bar{\text{x}}} \pm {\text{s}}$, n = 6)

Parameters	Con. (μg/mL)	Duodenum	Jejunum	Ileum	Colon
P_eff_ × 10^−3^	Triptolide	5.2 ± 1.72	4.98 ± 1.67	6.46 ± 2.0	6.35 ± 1.28
	TW	8.5 ± 3.61	5.54 ± 1.54	7.87 ± 1.68	8.35 ± 1.17^#^
	TW-PN	4.2 ± 1.23*	4.3 ± 0.98	5.9 ± 0.86*	5.36 ± 0.75**
	TW-CS	6.8 ± 2.12	5.4 ± 1.77	6.68 ± 0.9	6.95 ± 0.72*
	QLTB	2.8 ± 0.54**	4.03 ± 1.55	5.28 ± 0.81**	5.73 ± 0.26**
10 cm%ABS	Triptolide	42.7 ± 8.51	41.3 ± 9.1	42.8 ± 6.64	41.1 ± 3.33
	TW	45.6 ± 5.83	42.5 ± 4.3	43.4 ± 2.33	47.8 ± 2.76^##^
	TW-PN	37.38 ± 6.47*	28.9 ± 3.5**	37.9 ± 1.67**	29.9 ± 5.32**
	TW-CS	38.38 ± 4.21*	30.5 ± 7.3**	42.5 ± 2.12	27.9 ± 5.09**
	QLTB	29.9 ± 4.33**	23.1 ± 2.1**	28.4 ± 3.17**	21.5 ± 3.19**
K_a_ × 10^−2^	Triptolide	1.79 ± 0.33	1.64 ± 0.39	2.93 ± 0.29	1.81 ± 0.11
	TW	1.83 ± 0.82	2.63 ± 0.47^##^	2.31 ± 0.30^##^	1.95 ± 0.20
	TW-PN	0.93 ± 0.29*	1.34 ± 0.11**	1.76 ± 0.45*	1.71 ± 0.36
	TW-CS	1.19 ± 0.56	1.11 ± 0.25**	2.18 ± 0.35	1.55 ± 0.26*
	QLTB	1.27 ± 0.395	0.88 ± 0.25**	1.52 ± 0.21**	1.15 ± 0.22**

^#^*p* < 0.05, ^##^ *p* < 0.01, compared with triptolide; * *p* < 0.05, ** *p* < 0.01, compared with TW

### Absorption properties of triptonide in intestinal tract

Similar with triptolide, the absorption parameters, P_eff_, 10 cm%ABS and K_a_ showed concentration-independent characteristics within range of 3–9 μg/mL, and none significant variation of P_eff_ and 10 cm%ABS was observed among the four intestinal segments, duodenum, jejunum, ileum and colon. But the highest K_a_ was observed in the duodenum (Table 4). Unlike triptolide, TW did not promote obviously the absorption of triptonide. The P_eff_, 10 cm%ABS and K_a_ were pronouncedly reduced in the formulae of TW-PN and QLTB within the lumen of duodenum, jejunum, ileum and colon, and QLTB exerted more influence than TW-PN. Moreover, the K_a_ of triptonide was down-regulated significantly in the duodenum and jejunum (Table 5).

**Table 4:** Absorption parameters of triptonide in perfusate solution within duodenum, jejunum, ileum and colon (${\bar{\text{x}}} \pm {\text{s}}$, n = 6)

Parameters	Con. (μg/mL)	Duodenum	Jejunum	Ileum	Colon
P_eff_ × 10^−3^	3	11.6 ± 2.95	8.77 ± 1.47	9.34 ± 2.92	8.40 ± 1.94
	6	10.9 ± 3.55	8.92 ± 0.97	9.72 ± 2.41	8.52 ± 1.78
	9	12.3 ± 2.31	8.84 ± 0.96	9.81 ± 3.44	8.32 ± 1.18
10 cm%ABS	3	46.1 ± 2.89	44.9 ± 3.67	44.5 ± 3.31	45.6 ± 3.76
	6	43.9 ± 4.52	46.4 ± 2.49	43.8 ± 3.21	46.5 ± 3.07
	9	44.7 ± 3.78	46.1 ± 2.98	45.4 ± 1.99	45.7 ± 3.76
K_a_ × 10^−2^	3	5.64 ± 1.22	2.60 ± 0.23	1.15 ± 0.47	1.33 ± 0.66
	6	5.49 ± 1.42	2.48 ± 0.42	1.08 ± 0.52	1.28 ± 0.57
	9	5.57 ± 1.35	2.55 ± 0.33	1.16 ± 0.38	1.31 ± 0.63

**Table 5:** Absorption parameters of triptonide of various formulae in perfusate solution within duodenum, jejunum, ileum and colon (${\bar{\text{x}}} \pm {\text{s}}$, n = 6)

Parameters	Con. (μg/mL)	Duodenum	Jejunum	Ileum	Colon
P_eff_ × 10^−3^	Triptonide	11.6 ± 2.95	8.77 ± 1.47	9.34 ± 2.92	8.40 ± 1.94
	TW	13.3 ± 3.43	8.98 ± 1.26	11.3 ± 3.3	10.4 ± 2.04
	TW-PN	7.86 ± 1.36**	6.12 ± 1.38**	7.05 ± 1.29*	6.6 ± 1.57**
	TW-CS	12.8 ± 1.28	7.68 ± 1.64	9.93 ± 2.73	8.44 ± 1.31
	QLTB	5.55 ± 0.55**	5.48 ± 1.07**	4.75 ± 1.18**	5.22 ± 1.13**
10 cm%ABS	Triptonide	46.1 ± 2.89	44.9 ± 3.67	44.5 ± 3.31	45.6 ± 3.76
	TW	51.2 ± 8.5	46.8 ± 5.76	47.7 ± 7.12	49.7 ± 4.5
	TW-PN	40.4 ± 4.7*	33.6 ± 3.04**	37.0 ± 6.96*	35.9 ± 5.8**
	TW-CS	46.8 ± 5.3	41.3 ± 4.09	44.4 ± 4.18	45.6 ± 3.1
	QLTB	34.0 ± 5.7**	31.9 ± 3.27**	31.1 ± 7.18**	33.8 ± 3.4**
K_a_ × 10^−2^	Triptonide	5.64 ± 1.22	2.60 ± 0.23	1.15 ± 0.47	1.33 ± 0.66
	TW	6.03 ± 1.03	2.81 ± 0.18	1.32 ± 0.55	1.53 ± 0.46
	TW-PN	4.51 ± 0.83*	1.86 ± 0.14**	1.47 ± 0.68	0.99 ± 0.33*
	TW-CS	2.9 ± 1.68**	1.46 ± 0.28**	1.23 ± 0.78	1.37 ± 0.49
	QLTB	3.13 ± 1.11**	1.42 ± 0.08**	1.39 ± 0.36	0.70 ± 0.13**

* *p* < 0.05, ** *p* < 0.01, compared with TW

### Absorption properties of wilforlide in intestinal tract

With the enhancement of concentrations in the perfusate, P_eff_, 10 cm%ABS and K_a_ of wilforlide were retained constant relatively, which indicated the passive transporting and/or unsaturated active absorption process. The cardinal absorption locations of wilforlide were jejunum and ileum (Table 6). TW elevated wilforlide absorption within the intestinal tract, and significant enhancement of P_eff_ of wilforlide in TW was observed in jejunum and colon.

**Table 6:** Absorption parameters of wilforlide in perfusate solution within duodenum, jejunum, ileum and colon (${\bar{\text{x}}} \pm {\text{s}}$, n = 6)

Parameters	Con. (μg/mL)	Duodenum	Jejunum	Ileum	Colon
P_eff_ × 10^−3^	3	5.49 ± 2.14	9.57 ± 1.25	12.2 ± 3.14	9.65 ± 1.95
	6	5.25 ± 2.08	9.93 ± 0.78	12.5 ± 2.97	9.35 ± 2.13
	9	5.17 ± 2.69	10.2 ± 1.14	11.7 ± 3.26	10.7 ± 1.89
10 cm%ABS	3	31.3 ± 3.26	37.5 ± 2.47	41.3 ± 3.78	42.1 ± 4.22
	6	29.8 ± 3.89	37.9 ± 2.34	43.1 ± 3.27	45.7 ± 1.63
	9	30.6 ± 2.75	39.5 ± 0.64	40.3 ± 4.26	41.6 ± 4.64
K_a_ × 10^−2^	3	1.34 ± 0.55	2.27 ± 0.21	2.53 ± 0.43	1.36 ± 0.12
	6	1.57 ± 0.33	2.21 ± 0.15	2.48 ± 0.56	1.27 ± 0.26
	9	1.44 ± 0.48	2.27 ± 0.20	2.61 ± 0.19	1.14 ± 0.43

Both TW-PN and TW-SC decreased the absorption extent and rate of wilforlide, especially in the jejunum, ileum and colon (p < 0.05). However, wilforlide was not detected in QLTB perfusate prepared according to the ratio of herbal medicines (Table 7).

**Table 7:** Absorption parameters of wilforlide of various formulae in perfusate solution within duodenum, jejunum, ileum and colon (${\bar{\text{x}}} \pm {\text{s}}$, n = 6)

Parameters	Con. (μg/mL)	Duodenum	Jejunum	Ileum	Colon
P_eff_ × 10^−3^	Wilforlide	6.07 ± 2.14	9.57 ± 1.25	12.2 ± 3.14	9.65 ± 1.95
	TW	6.69 ± 1.93	11.6 ± 1.08^#^	14.7 ± 3.51	12.5 ± 1.65^#^
	TW-PN	5.91 ± 1.03	4.41 ± 1.27**	5.46 ± 2.79**	9.9 ± 0.63**
	TW-CS	4.98 ± 1.39	3.89 ± 0.41**	10.6 ± 1.27*	4.77 ± 2.56**
	QLTB	NA	NA	NA	NA
10 cm%ABS	Wilforlide	31.3 ± 3.26	37.5 ± 2.47	41.3 ± 3.78	42.1 ± 4.22
	TW	33.2 ± 7.06	39.8 ± 1.51	43.5 ± 4.44	44.0 ± 3.51
	TW-PN	26.2 ± 5.75	18.2 ± 5.69**	21.4 ± 1.35**	26.0 ± 4.69**
	TW-CS	26.8 ± 5.08	25.6 ± 4.16**	35.6 ± 1.37**	30.7 ± 1.59**
	QLTB	NA	NA	NA	NA
K_a_ × 10^−2^	Wilforlide	1.34 ± 0.55	2.27 ± 0.21	2.53 ± 0.43	1.36 ± 0.12
	TW	1.54 ± 0.55	2.39 ± 0.03	2.73 ± 0.35	1.54 ± 0.19
	TW-PN	1.37 ± 0.32	0.92 ± 0.44**	1.18 ± 0.78**	0.83 ± 0.39**
	TW-CS	1.18 ± 0.32*	0.77 ± 0.15**	1.21 ± 0.54**	0.50 ± 0.33**
	QLTB	NA	NA	NA	NA

^#^*p* < 0.05, compared with wilforlide; * *p* < 0.05, ** *p* < 0.01, compared with TW; *NA* none detected

### Absorption properties of tripterine in intestinal tract

Sharing the possible same transport process with the above three chemicals, P_eff_, 10 cm%ABS and K_a_ of tripterine were independent of concentration in the perfusate. Although tripterine had greater K_a_ in jejunum and ileum than that in duodenum and colon, minor fluctuation of P_eff_ and 10 cm%ABS between the four intestinal segments (Table 8). As shown in Table 9, TW facilitated the absorption of tripterine partly. But significant elevation of absorption extent and rate of tripterine was observed in TW-PN, which demonstrated that PN further augment the effect of TW. However, TW-SC inhibited the absorption of tripterine in the intestine suggested SC dramatically reversed the action of TW on the absorption of tripterine. Interestingly, QLTB demonstrated promotion of tripterine absorption in the duodenum and blockage in the ileum and colon.

**Table 8:** Absorption parameters of tripterine in perfusate solution within duodenum, jejunum, ileum and colon (${\bar{\text{x}}} \pm {\text{s}}$, n = 6)

Parameters	Con. (μg/mL)	Duodenum	Jejunum	Ileum	Colon
P_eff_ × 10^−3^	3	7.11 ± 3.68	8.72 ± 3.47	9.48 ± 2.91	10.3 ± 1.56
	6	6.93 ± 3.01	9.52 ± 3.28	8.15 ± 3.82	10.8 ± 1.79
	9	9.03 ± 1.35	10.5 ± 1.67	9.13 ± 2.63	8.57 ± 3.75
10 cm%ABS	3	4.76 ± 1.27	4.98 ± 2.46	5.62 ± 1.81	6.34 ± 1.63
	6	4.69 ± 1.46	5.19 ± 1.44	5.34 ± 2.57	5.59 ± 2.34
	9	4.75 ± 0.98	5.09 ± 1.45	5.46 ± 2.24	5.75 ± 1.86
K_a_ × 10^−2^	3	1.52 ± 0.41	2.08 ± 0.33	2.62 ± 0.17	1.27 ± 0.19
	6	1.66 ± 0.23	2.12 ± 0.23	2.48 ± 0.45	1.03 ± 0.37
	9	1.44 ± 0.58	2.03 ± 0.45	2.43 ± 0.74	1.19 ± 0.24

**Table 9:** Absorption parameters of tripterine of various formulae in perfusate solution within duodenum, jejunum, ileum and colon (${\bar{\text{x}}} \pm {\text{s}}$, n = 6)

Parameters	Con. (μg/mL)	Duodenum	Jejunum	Ileum	Colon
P_eff_ × 10^−3^	Tripterine	6.78 ± 3.68	8.72 ± 3.47	9.48 ± 2.91	10.3 ± 1.56
	TW	7.11 ± 2.77	10.7 ± 4.51	11.6 ± 3.01	12.4 ± 3.63
	TW-PN	10.3 ± 1.14*	13.3 ± 4.08	15.2 ± 2.63	9.98 ± 0.21
	TW-CS	3.20 ± 0.49**	3.35 ± 0.78**	5.69 ± 2.12**	3.39 ± 1.15**
	QLTB	11.7 ± 0.82**	11.1 ± 5.3	6.10 ± 3.63*	5.62 ± 3.11**
10 cm%ABS	Tripterine	4.76 ± 1.27	4.98 ± 2.46	5.62 ± 1.81	6.34 ± 1.63
	TW	5.87 ± 1.23	6.27 ± 2.26	7.60 ± 1.17^#^	7.14 ± 1.63
	TW-PN	6.49 ± 1.91	9.29 ± 0.54**	9.43 ± 0.56**	5.82 ± 1.42
	TW-CS	2.91 ± 0.49**	3.74 ± 0.95*	4.23 ± 2.67*	3.97 ± 1.19**
	QLTB	8.13 ± 0.81**	7.38 ± 1.07	4.84 ± 1.58**	5.65 ± 1.31
K_a_ × 10^−2^	Tripterine	1.52 ± 0.41	2.08 ± 0.33	2.62 ± 0.17	1.27 ± 0.19
	TW	1.88 ± 0.38	2.21 ± 0.51	2.91 ± 0.35	1.34 ± 0.54
	TW-PN	2.31 ± 0.83	3.27 ± 0.21**	3.21 ± 0.14	2.52 ± 0.13**
	TW-CS	1.64 ± 0.30	1.22 ± 0.20**	1.39 ± 0.40**	1.54 ± 0.11
	QLTB	2.14 ± 0.30	1.69 ± 0.45	1.86 ± 0.16**	1.66 ± 0.31

^#^*p* < 0.05, compared with tripterine; * *p* < 0.05, ** *p* < 0.01, compared with TW

## Discussion

Oral administration is definitely the most common and convenient route in the therapeutic system of traditional Chinese medicine and natural herb. The absorption extent and rate, namely bioavailability, at which unchanged drug proceeds from the gastrointestinal tract to the system circulation successively passing through the apical membrane of the epithelial cells, pre-hepatic blood vessels, portal vein and liver is the first limiting step of the pharmacokinetic process, which directly influences the peak concentrations of drug and retention time in the body and closely involves in the drug actions and side effect [13, 14]. It’s paramount to evaluate the effective bioavailability of screening drug candidates in developing an oral dosage form [15]. Absorption is a complex kinetic process that is dependent on numerous factors including the physiochemical properties of the drug candidates and the physiochemical properties of the gastrointestinal barrier membrane [16]. During the chemicals passing through the gastrointestinal tract, solubility is a critical parameter for absorption since they must be in solution to permeate the intestinal wall, which is influenced by the ionization, molecular weight and lipophilicity. Therefore, introducing ionizable groups, reducing molecular size and lipophilicity are the most efficient and frequent strategy used by medicinal chemists to increase the solubility. The p*K*a of molecules and the pH of gastrointestinal tract jointly govern the solubility. The above mentioned properties are the primarily limiting factors of the passive transport, the driving force of which is the concentration gradient between the two sides of the membrane, allowing chemicals to move from the side of higher concentration to the side of lower concentration without the expenditure of cellular energy. Moreover, the carriers-mediated active transport requires specialized carrier proteins allowing chemicals to cross the membrane against a concentration gradient [17–19]. According to the prescription principal, the oral formulation of decoction from single or several combinational herbs is the routine remedy regimen carrying some active components through the gastrointestinal tract. Unlike chemical drug candidates, there are no directly artificial modifications of chemical structure in the herbal remedy. Similar with chemical drugs, however, the components of herb are undergoing the permeation process of influx and efflux through the intestinal membrane. The components from the same herb or these compatible ones are responsible for regulating absorption circumstance such as the pH and involved transporters [20].

Component-component interaction is the basic characteristics of the decoction formulation during the absorption process within the gastric and intestinal lumen, at or within the gut wall, as well as within the liver, which leads to the effects of the potentiation or antagonism between them [21–23]. One of the common mechanisms underlying the interaction is the alteration of gastrointestinal pH. The solubility of drug with specific pKa is regulated by the intestinal fluid pH. Basic drugs are more soluble in acidic fluids, and acidic drugs are more soluble in basic fluids. However, drug solubility does not completely ensure absorption because only un-ionized molecules are absorbed. Tannin, one kind of plant polyphenol, is widespread in leaves, roots and fruits of plant, has the ability to change the pH of gastrointestinal tract. Tannic acid is a polymer of gallic acid molecules and glucose, and demonstrates weak acidity due to the numerous phenol groups in the structure. In the decoction of herbal medicine, tannin binds with other components such as alkaloids forming chelation and directly changing the solubility of some active chemicals, which results in failing to permeate the intestinal mucosa [24, 25]. However, acidic chemicals like tannin are verified to inhibit the motility of intestine, which prolong the exposure period and might enhance the absorption [26]. Both TW and PN contain tannin. Another mechanism possibly influencing the absorption of the four components of TW investigated in the present study is ameliorating intestinal blood flow by PN theoretically affecting the absorption of lipophilic compounds. Notoginseng triterpenes from PN shows positive effect on the cerebral hypoxia and myocardial ischemia via dilating the blood vessels [27]. The vasoactive action can still be held on the mesentery. Meanwhile, monomers Rg3 in the notoginseng triterpenes inhibited the P-glycoprotein (P-gp) function and promoted accumulation of rhodamine 123 in drug-resistant KBV20C cells in a dose-dependent manner [28]. P-gp is an efflux transporter proteins from the ATP-binding cassette family expressing at the lumenal surface of the intestinal epithelium and opposing the absorption by transporting lipophilic compounds out of enterocytes back into the gastrointestinal lumen [29, 30]. Interestingly, some specific substrates of P-gp have affinities to a pivotal metabolism enzyme CYP3A responsible for Phase I oxidative metabolism [31]. Generally, inhibition of CYP3A4/5 results in a minimum threefold increase in the extent of absorption and toxicity of the concomitantly administered agent, but can also result in decreased efficacy of prodrugs needing CYP3A for conversion to active moieties. Notoginseng triterpenes was demonstrated to inhibit the CYP3A in liver, and the action on the CYP3A4/5 in intestine is supposed [32]. However, further investigations are required to suggest which component associated with the toxicity is the substrates of CYP3A4/5.

SPIP is a typical in situ intestinal perfusion model to study absorption rates [10]. Although the animal has been anaesthetized and surgically manipulated, the neural, endocrine, lymphatic, and mesenteric blood supplies are still working and therefore all the transport mechanisms are maintained, which is more advantage than in vitro techniques. Meanwhile, multiple samples may be taken, thus enabling kinetic studies to be performed in situ intestinal perfusion method, data from which at the rat model has been demonstrated to correlate with in vivo human data [33]. Unlike the closed loop intestinal perfusion technique, SPIP is an open loop system that is developed to evaluate the properties of drug absorption with continuous fluid flow through the intestine and provides better control of the hydrodynamics and increased surface area than the closed loop method. Although the P_eff_ values are generally similar obtained from both open and closed loop techniques, SPIP is proven to produce more reproducible absorption rat and lower variance within experiments. Otherwise, what should be considered for the SPIP is the assumption that all drug passes into portal vein, that is drug disappearance reflects drug absorption, may not be valid in some circumstances. The bio-transformations in intestine by the major cytochrome P450 enzymes, CYP3A4/5, is also responsible for the disappearance of in intestinal lumen and significantly reduce oral bioavailability [34, 35]. Then the absorption rate estimated in SPIP should be called disappearance rate. The technique to overcome the shortcoming of SPIP is intestinal perfusion with venous sampling models. Based on the appearance kinetics in pre-hepatic blood, drug absorption through the enterocytes can be quantified through plasma sample analysis, which facilitates quantification of both steady-state disappearance kinetics from the intestinal lumen and concurrent appearance kinetics into pre-hepatic blood. The drug appearance in pre-hepatic blood represents the net levels of absorption into the apical membrane and flux through the enterocyte. For compounds with minimal intestinal first-pass metabolism, the P_eff_ calculated with the disappearance from lumen and then appearance into pre-hepatic blood is similar [36]. Moreover, the experimental operation of SPIP is simpler than the intestinal perfusion with plasma sampling.

P_eff_ is the effective intestinal permeability coefficient and commonly used to estimate the extent of absorption, which is proportional to the first-order absorption rate constant, K_a_, and weighted with the surface area and the volume of the intestine [37, 38]. The estimation of P_eff_ may be impacted by several factors such as reperfusion flow rates, intestinal radius, intestinal surface area and the time to reach steady-state conditions. In SPIP model, the most widely used estimate for the rat intestinal radius is 0.18 cm/min. It was maintained 0.2 cm/min in the present study and the length of intestine used to estimate P_eff_ is 10 cm, which make consistent of the radius and surface area of intestine. The value of P_eff_ represents the extent and rate of absorption and also be employed to distinguish the transport process of chemical. With the increasing drug concentration in the perfusate, constant or no significant variation of P_eff_ value demonstrates the passive diffusion, a reduction in P_eff_ suggests saturation of carrier-mediated influx, and an increasing P_eff_ values hints saturation of efflux transporters. In this study, the P_eff_ of triptolide, triptonide, and wilforlide at different concentration are consistent in the duodenum, jejunum, ileum and colon, respectively. Similar conclusion aroused from tripterine, but in duodenum and jejunum, P_eff_ of tripterine has the increasing tendency (p > 0.05). These four components of TW should be absorbed in the rat intestine through passive diffusion primarily or unsaturated carrier-mediated absorption. Furthermore, no obvious difference of P_eff_ was observed between the small intestine and colon.

## Conclusions

In the present investigation, four ingredients were employed to reveal the absorption process of TW in the absence or presence of other herbal materials, which help to understand the basic principle of compatibility as action promotion and toxicity neutralization. In general, these interesting chemicals in TW show quicker absorption rate and greater influx extent than the pure ones, which lead to the increased therapeutic effect and the paralleled adverse reaction as well. For triptolide, triptonide and wilforlide, the combinational formulae decreased their absorption. Synergic action was observed in QLTB, which exhibit the most powerful influence. However, intestinal absorption of tripterine with relative therapeutic window was enhanced in the formula of TW-PN indicated PN promoted TW-derived tripterine influx. But in formula of TW-SC, SC still block the absorption process of tripterine. Opposite-direction of PN and SC finally resulted in the different appearance of tripterine absorption through the four intestinal segments in QLTB as increased in duodenum and jejunum, and decreased in ileum and colon.

In summary, herbal compatibility regulates the intestinal absorption characteristics of TW-containing chemicals, which is responsible for the promoting therapeutic effect and reducing toxicity. In the formula QLTB, PN is the key subsidiary component to support the sovereign medicinal to complete the treatment. As shown in this study, one of pivotal mechanism underlying the positive role of PN and QLTB is modulating the absorption process in the intestinal lumen.

## Additional file

**Additional file 1.** Minimum Standards of Reporting Checklist.

## Abbreviations

10 cm%ABS: 10 cm intestinal absorption
CS: *Caulis Sinomenii*
QLTB: Qing-Luo Tong-Bi
QC: quality control
P_eff_: permeability coefficient
P-gp: P-glycoprotein
PN: *Panax notoginseng*
RSD: relative standard deviation
SD: standard deviation
SPIP: single-pass intestinal perfusion
TW: *Tripterygium wilfordii*
UPLC-MS/MS: ultra-performance liquid-chromatography tandem mass spectrometry
ALT: alanine aminotransferase
AST: aspartate aminotransferase
LDH: lactate dehydrogenase
